# Advancing Digital Health Innovation in Oncology: Priorities for High-Value Digital Transformation in Cancer Care

**DOI:** 10.2196/43404

**Published:** 2023-01-04

**Authors:** Smit Patel, Jennifer C Goldsack, Grace Cordovano, Andrea Downing, Karen K Fields, Cindy Geoghegan, Upinder Grewal, Jorge Nieva, Nikunj Patel, Dana E Rollison, Archana Sah, Maya Said, Isabel Van De Keere, Amanda Way, Dana L Wolff-Hughes, William A Wood, Edmondo J Robinson

**Affiliations:** 1 Digital Medicine Society Boston, MA United States; 2 Enlightening Results LLC West Caldwell, NJ United States; 3 The Light Collective Shoreline, WA United States; 4 Center for Digital Health Moffitt Cancer Center Tampa, FL United States; 5 Patient and Partners LLC Madison, CT United States; 6 Bayer AG Reading United Kingdom; 7 Norris Comprehensive Cancer Center University of Southern California Los Angeles, CA United States; 8 AstraZeneca PLC Gaithersburg, MD United States; 9 AS Pharma Advisors, Inc San Francisco, CA United States; 10 Outcomes4Me Inc Boston, MA United States; 11 Immersive Rehab London United Kingdom; 12 Jazz Venture Partners San Francisco, CA United States; 13 Division of Cancer Control and Populations Sciences National Cancer Institute Bethesda, MD United States; 14 Lineberger Comprehensive Cancer Center University of North Carolina Chapel Hill Chapel Hill, NC United States

**Keywords:** digital health, innovation, oncology, cancer care, cancer, patient journey, digital transformation, digital divide, health care delivery, service delivery, equity, patient-reported outcome, PROM, biomarker, digital innovation

## Abstract

Although health care delivery is becoming increasingly digitized, driven by the pursuit of improved access, equity, efficiency, and effectiveness, progress does not appear to be equally distributed across therapeutic areas. Oncology is renowned for leading innovation in research and in care; digital pathology, digital radiology, real-world data, next-generation sequencing, patient-reported outcomes, and precision approaches driven by complex data and biomarkers are hallmarks of the field. However, remote patient monitoring, decentralized approaches to care and research, “hospital at home,” and machine learning techniques have yet to be broadly deployed to improve cancer care. In response, the Digital Medicine Society and Moffitt Cancer Center convened a multistakeholder roundtable discussion to bring together leading experts in cancer care and digital innovation. This viewpoint highlights the findings from these discussions, in which experts agreed that digital innovation is lagging in oncology relative to other therapeutic areas. It reports that this lag is most likely attributed to poor articulation of the challenges in cancer care and research best suited to digital solutions, lack of incentives and support, and missing standardized infrastructure to implement digital innovations. It concludes with suggestions for actions needed to bring the promise of digitization to cancer care to improve lives.

## Introduction

In an ideal world, the incidence and disease burden of cancer would be reduced and inequities ameliorated by improvements in education, prevention, screening, and early detection. Every person living with cancer could easily access timely screening and the right treatment at the right time. Personalized treatment would be the standard of care, and patients’ health status would be continually monitored to ensure their safety and optimize their quality of life. In addition, patients and their care partners [1] would have ready access to high-quality research and fit-for-purpose educational resources to help them understand their diagnosis and care journey. Care partners would be welcomed into the lifetime journey that a cancer diagnosis or prognosis can bring, and patients whose lives are cut short by cancer would be supported with access to high-quality end-of-life care rooted in dignity and compassion. Additionally, clinicians could follow their vocation to save and improve lives, with documentation, reimbursement, and payment cycle workflows fully optimized to reduce their administrative burden.

In the digital era of health care, it is possible for data and information to flow freely and for patient-physician encounters to transcend place and time. Currently, however, cancer care is incredibly frustrating and physically, mentally, emotionally, and financially taxing for patients, care partners, and clinicians alike, and this burden is not distributed equally [2]. The likelihood of survival from cancer varies by the patient’s zip code [3], race [4-8], socioeconomic status [9,10], sex [11,12], and site of care [13]. Patients who *do* survive cancer can face the long-term effects of physical and financial toxicity [14-16]. Cancer care is disconnected from cancer research, preventing refinement of care from each test performed, diagnosis made, and treatment administered. Disparate data sources and systems hamper coordination of care and raise the possibility of gaps in management or even working at cross purposes.

The integrated digitization of the various streams of health care data can inform better decision-making [17-26] and improve coordination of care while respecting patient preference and privacy. Screening, diagnosis, and treatment can be informed by personalized risk models that apply equally well to every member of the population, in real time and on a continuous basis. For clinicians, integrated digitization offers the possibilities of streamlined administrative tasks and comprehensive views of their patients’ journeys, improving delivery of care. For payers, digitization can improve the efficiency of reimbursements and reduce the possibility of duplicative or redundant services and claims.

In December 2021, the Digital Medicine Society and Moffitt Cancer Center convened over 40 multistakeholder experts, including patients, clinicians, researchers, regulators, payers, health care executives, policy makers, technology innovators, investors, and advocates for presentations and a roundtable discussion. Conversations addressed the current state of digital innovation in cancer care, identified root causes of challenges to digital health innovation in oncology, and described action-oriented approaches to collaborative solutions to be advanced by the workshop hosts and partners. This viewpoint summarizes these discussions and suggests avenues for future exploration.

## The Digitization of Cancer Care: Opportunities and Obstacles

Cancer care is complex, often characterized by multiple interventions provided by a variety of providers over extended periods of time. This results in a high patient burden in the absence of support through care coordination. Additionally, oncology clinical pathways, intended to manage this complexity and support evidence-based care, improve outcomes, and contain costs, are not comprehensive enough and universally applied, leading to less-than-optimal clinical interventions despite clinicians’ best intentions and efforts.

Effective cancer treatment, management, and research require the facile exchange of large amounts of data and information. But standardization of oncology data is impeded by how we measure disease progression, with the most important information often buried in images, narratives, and other unstructured data. The interoperability challenges that characterize our current health system have an outsized negative impact on patients with cancer due to the volume and complexity of oncology data. Patients often face delays in accessing essential cancer care due to challenges in accessing and aggregating all of their necessary medical records and health information. This forces patients with cancer and their care partners to do substantial work to get the care they need; it also can affect their treatment outcomes [27,28].

Patients with cancer and their care partners also need better, more accessible, and culturally competent information to better navigate their disease. Currently, most patients lack access to point-of-care educational materials designed to help them navigate the decisions they must make and access the emotional support they need as their disease evolves, resolves, or progresses. This includes end-of-life care. This unmet need for information is often compounded, particularly in the United States, by financial toxicity [29]—the problems a patient with cancer has related to the costs of their treatment—leaving significant gaps between our potential to care for people with cancer, their outcomes, and the reality of their lifetime cancer journeys.

## Target Digital Innovation to Do the Greatest Good for Individuals With Cancer

Fit-for-purpose digital innovation offers the potential to bridge the gap between the reality of cancer care today and our vision for caring for people with cancer in the future. Attendees of the roundtable agreed that digital innovation in oncology lags behind other therapeutic areas despite high unmet need. This lag was attributed to poor articulation of the challenges in cancer care and research best suited to digital solutions (Table 1), lack of incentives and support, and missing standardized infrastructure to implement digital innovations. We address these issues in the remainder of this piece.

**Table 1:** Persistent challenges in oncology that are highly suited for effective digital solutions.

Theme areas and topic			Challenges		Opportunities for digital innovation
**Research and development**					
	End point measurement	Current *performance status measures* in oncology are subjective, episodic, and provide limited high-value information about a patient		Define and optimize a set of *core digital measures* that address unmet clinical need by accelerating the development of high-quality, relevant, trustworthy digital measures of performance status	
	QoL^a^ measurement	Lack of *data on the impact of treatments on QoL* impedes optimal treatment decision-making		Develop digital *approaches to measuring the impact* of treatments on QoL	
	Scientific methods	*Lack of scientific methods* for evaluating digital solutions within the highly complex field of oncology		Develop and drive the broad adoption of standardized methodological approaches for digital oncology solutions	
**Clinical care**					
	Operational inefficiency	*Operational burden* associated with poorly coordinated clinical pathways in oncology has created ineffectiveness in care delivery		Leverage digital tools to *automate, augment, and streamline clinical operations* and decision support, making oncology care efficient so that dollars can be redeployed into direct care	
	Implementation barriers	Lack of clinician and health system confidence in digital innovation due to the *poorly implemented digital strategies* to date		Propose *implementation parameters* for deploying digital tools to support successful implementation in clinical care	
	Constrained capacity	Specialized *oncology workforce shortages and administrative burdens* delay necessary critical care [30,31]		*Modernize cancer care delivery* through *digitized capabilities* that reduce clinician administrative burden and free up capacity to provide timely necessary care to individuals with cancer	
**Care personalization and coordination**					
	Data integration	Myriad data sources generating *heterogeneous data* relevant to each patient’s cancer journey impede optimized decision-making		Develop approaches to *data integration* that streamline and aggregate various data sets and touchpoints for precision insights and clinical decision-making	
	Data governance	Data liquidity is limited due to the *lack of governance models* to support trustworthy data exchange and authorized access		Develop frameworks [32,33] and *approaches for increasing data liquidity* through rapid, seamless, and transparent sharing and exchange of data for personalized cancer care	
	Inclusion	*Persistent inequities* in cancer risk, disease incidence and burden, and clinical outcomes		Deploy *inclusive approaches* to all digital solutions: leverage new digital solutions to access hard-to-reach and underserved populations and capture data on populations who have been previously invisible	
**Payer policy and reimbursement**					
	Financial toxicity	Widespread treatment-related *financial harm* [29]		Prioritize reducing barriers to financial *assistance and digitize* traditionally paper-based *applications* to minimize processing and review times	
	Payer innovation	Inadequate systemwide *payment structures* [34-36] inhibit development and adoption of digital solutions		Build evidence for payment innovation with the development of *novel incentive structures* to implement digital health technologies favoring value-based care	
**Engagement and education**					
	Engagement	Challenges dedicating resources to consistent, active, and *equitable engagement* of populations relative to their cancer risks or disease burden [37]		Build clear broadly accepted *strategies* for ongoing equitable *engagement and re-engagement* of patients at various stages of their cancer journey with the use of digital tools	
	Education	*Lack of resources* for patients and care partners to navigate treatment decision-making negatively impacts the care of individuals with cancer [38]		Use digital approaches to make culturally appropriate *education materials* accessible to all individuals who could benefit	

^a^QoL: quality of life.

Effective digital solutions must focus on the full spectrum of each person’s cancer journey, from reducing the risk of diagnosis to whole-person support of every individual with a cancer diagnosis, not just episodic care clinically focused on treating cancer once diagnosed. Digital solutions must be evaluated in a longitudinal manner, recognizing that their value may change with disease progression and a lifetime of different needs. Digital solutions must be created that are valuable and acceptable to every person, recognizing that every patient with cancer deserves culturally competent care. We also must recognize the needs of care partners throughout patients’ cancer care journeys. There is no exception for these needs during advanced stages of disease and end-of-life care.

To place individuals living with cancer at the center of their care, digital solutions that support both privacy and information sharing should be prioritized. The health care delivery model should be reimagined, decentralizing it and moving the focus from the provider-centric facility-based health care model to a whole-person approach that meets patients wherever they may be.

Digitized decision-support systems offer the potential to deliver on the promise of oncology clinical pathways to improve outcomes and reduce costs. Further, they offer the possibility of removing disparities in outcomes between centers of excellence and community-based care settings by democratizing optimized decision-making. Tools that streamline data management in electronic health records and offer computerized clinical workflows could improve outcomes, efficiency, and provider satisfaction while minimizing the risk of harm. Addressing the needs and preferences of health care practices and clinicians must be considered integral to the success of any digital innovation and may be a target for many digital solutions.

To use research and development as an example, consider the use of biometric monitoring technologies. Before these technologies can be deployed in clinical research or practice, we must first consider the potential context of use. From the relevant health concept of interest identified, investigators can then develop potential metrics needed to measure this concept at baseline and over time. For geriatric oncology, contexts of use might include baseline frailty or electronic rapid fitness assessments and monitoring for adverse events or functional deterioration between clinic visits. The use of home-based electronic patient-reported outcomes monitoring is currently being evaluated for the latter purpose [39].

Whatever the outcomes used, they must be meaningful to patients. Cancer cachexia, for example, affects quality of life, treatment options, and mortality. Physical activity has been proposed as an outcome measure in cancer cachexia trials [40], but the gold standard for measurement of physical activity (eg, stable isotope studies) can be complex, expensive, and burdensome for patients. Digital health technologies are likely to offer advantages in situations such as this. In fact, a feasibility study showed near-universal acceptability to patients with cancer in wearing a small lightweight step monitor for a week [41]. The investigators noted strong correlations between step counts and both stepping and nonstepping energy expenditure [41]. Physical activity has also been shown to be correlated with quality-of-life scores in patients with advanced cancer [42]. In addition, nutraceutical intervention has been shown to improve physical activity in patients with cachexia due to pancreatic cancer [43].

The use of digital technologies also offers an opportunity to increase the enrollment of patients with cancer into clinical trials. In a survey of more than 1100 patients who had been diagnosed with or treated for cancer in the past 7 years, up to 85% stated they would be more likely to enroll in clinical trials if the burdens on their time and travel were reduced, including through the use of remote technology and other tools to decentralize data gathering [44]. Their willingness varied depending on the type of decentralization: 67% said they would be more likely to enroll if offered intravenous trial medications administered by trial staff, whereas 82% said they would be more likely to sign up if they were offered wearable technology to gather trial data.

## Delivering on the Promise of Digital Innovation to Advance a Better Approach to Cancer Care

For digital innovation to deliver on its potential to improve the lives of all people with cancer, we must prioritize the opportunities for greatest impact and consider equity and inclusion as givens when developing and deploying digital solutions, acknowledge and address the methodological gaps and absence of data infrastructure required for successful digital health innovation, and recognize that digital innovation in cancer care and research is not a silver bullet; we have rich flows of data and a powerful new suite of tools in the toolbox, but absent a human-centered approach with a focus on access, equity, efficiency, and effectiveness, digital tools are unlikely to be solutions for this disease that claims the lives of one in six people worldwide.

The National Cancer Act was passed in 1971 and has driven waves of improvements in the lives of people diagnosed with cancer. Earlier this year, the US government reignited the cancer “moonshot” [45], noting that the death rate from cancer has decreased by 25% over the past 25 years. Among new initiatives the administration touted were the National Institutes of Health (NIH) expansion of the Cancer Research Data Ecosystem, which supports better data sharing for treatment discovery [46]. The president also announced a new proposed entity within the NIH, the Advanced Research Projects Agency for Health. This agency, modeled after the Department of Defense’s Advanced Research Project Agency, will have a singular purpose: to drive breakthroughs to prevent, detect, and treat diseases, including cancer [45]. The US Department of Agriculture is also working with the NIH to combine persistent poverty and cancer surveillance data to identify targets for intervention [46]. But without the intentional embrace of digital solutions that work for all individuals, we are likely to fall short of the promise of patient-, clinician-, and system-level transformation that well-implemented fit-for-purpose digital innovations offer.

There is no clinical or technical reason for the successful digitization of oncology to lag behind other therapeutic areas. Digital innovation promises to support the development of more effective and equitable approaches to risk reduction, reduction of disease burden, accelerated research, and care that can scale. It is up to us, as a multistakeholder community, to identify the right problems in oncology to solve, to develop the science and infrastructure necessary to build trust, to support clinicians and practices through digital transformation with the appropriate resources, and to reimagine the systems of research and care necessary for digital approaches to truly transform the life of every person living with cancer.

## Abbreviations

NIH: National Institutes of Health
